# Lactate dehydrogenase A is a diagnostic biomarker associated with immune infiltration, m6A modification and ferroptosis in endometrial cancer

**DOI:** 10.3389/fonc.2024.1458344

**Published:** 2024-11-08

**Authors:** Yan Huang, Weichun Tang, Liping Chen

**Affiliations:** ^1^ Department of Gynaecology and Obstetrics, Affiliated Hospital 2 of Nantong University, Nantong First People’s Hospital, Nantong, China; ^2^ Department of Gynaecology and Obstetrics, Jianhu People’s Hospital, Jianhu, China

**Keywords:** lactate dehydrogenase A, endometrial cancer, immune infiltration, m6A modification, ferroptosis

## Abstract

**Background:**

Lactate dehydrogenase A (LDHA) has been confirmed as a tumor promoter in various cancers, but its role in endometrial cancer remains unclear.

**Methods:**

The Cancer Genome Atlas (TCGA), quantitative real-time polymerase chain reaction and the Human Protein Atlas were utilized to analyzed the LDHA expression in EC. The LDHA levels of patients with different clinical features were compared based on the TCGA cohort. The Genome Ontology, Kyoto Encyclopedia of Genes and Genomes, and Gene Set Enrichment Analysis of LDHA-related genes were conducted by R language. The influence of LDHA knockdown on cell proliferation, apoptosis, migration and invasion was detected by *in vitro* experiment. The relationship between LDHA expression and immune infiltration was explored by Tumor Immune Estimation Resource 2.0 and Gene Expression Profiling Interactive Analysis. The association of LDHA level with N6-methyladenosine (m6A) modification and ferroptosis was investigated based on the TCGA-UCEC and the GEO cohort.

**Results:**

The LDHA was overexpressed in EC tissues and EC cell lines, and had high predictive accuracy for the EC diagnosis. The LDHA level was associated with age, histological type, histologic grade, and radiation therapy. LDHA-related genes participated in multiple biological functions and signaling pathways. LDHA downregulation significantly promoted cell apoptosis and inhibited the proliferation, migration, and invasion of EC cells. LDHA expression was connected to multiple tumor-infiltrating lymphocytes (TILs), m6A-related genes, and ferroptosis-related genes.

**Conclusion:**

LDHA has the potential to work as an EC biomarker associated with TILs, m6A modification, and ferroptosis in EC.

## 1 Introduction

Endometrial cancer (EC) is the sixth most common malignancy in women, with 417 000 new confirmed cases and 97 000 deaths worldwide in 2020 (1). The EC incidence is still rising globally due to the influence of modern high-fat, high-calorie diets, and less-exercise lifestyles (2). Surgery is the primary treatment for EC, but radiotherapy, chemotherapy and targeted therapy are increasingly important in the EC treatment (3). With the continuous improvement of the medical level, the prognosis of EC patients has been dramatically improved, but the therapy effect on patients with high-risk factors or in the advanced stage remains unsatisfactory. It’s essential to explore the molecular pathogenesis of EC for seeking a new therapeutic breakthrough.

Lactate dehydrogenase A (LDHA) is an important glycolytic metabolic enzyme that converts pyruvate into lactic acid, increasing tumor cells’ glucose uptake and lactic acid production (4). As an vital enzyme index, LDHA has been shown to participate in the regulation of tumor formation, growth and metastasis. LDHA knockout significantly reduced the metastasis potential of hepatocellular carcinoma in xenograft mouse models (5). In renal cell carcinoma, LDHA knockdown promoted cell apoptosis and inhibited cell migration (6). LDHA interference also inhibited the occurrence and development of tumors through the mitochondrial pathway to induce breast cell apoptosis (7). Meanwhile, LDHA overexpression could promote the proliferation and differentiation of pancreatic cancer cells (8). However, little is known about LDHA expression and its potential role in EC.

This study aimed to expound the role of LDHA in EC progression, and explore the potential mechanism around LDHA. The study analyzed the data from The Cancer Genome Atlas (TCGA)-UCEC and the Gene Expression Omnibus (GEO) cohort. Bioinformatics methods were performed to detect the LDHA expression in EC tissues and normal tissues, and to explore the mechanism of LDHA in EC progression. The influence of LDHA on the malignant biological behavior of EC cells was also confirmed by *in vitro* experiments. The relationship between LDHA and immune infiltration, N6-methyladenosine (m6A) modification, and ferroptosis was demonstrated. We hope this study could provide a novel basis for EC therapeutic strategies.

## 2 Materials and methods

### 2.1 LDHA expression in EC tissues

The data of the TCGA-UCEC cohort were analyzed to compare the differential expressions in cancer tissues and normal tissues by Student’s t-test and paired t-test, respectively. The ROC curve was performed by the R package “pROC” and visualized using the package “ggplot2”. The protein expression of LDHA was confirmed by the Human Protein Atlas database (https://www.proteinatlas.org/).

### 2.2 Cell lines

The endometrial cells and EC cell lines Ishikawa, HEC-1-A, HEC-1-B and KLE were purchased from FuHeng Biology (Shanghai, China). Cells were cultured in an incubator with 37°C and 5% CO_2_. The culture medium was prepared as DMEM medium with 10% fetal calf serum (FBS) (#10099141C, Gibco, USA) and 1% penicillin-streptomycin (#15140148, Gibco, USA).

### 2.3 Quantitative real-time polymerase chain reaction

The total RNA was extracted from each cell by TRIzol reagent (#15596018CN, Invitrogen, USA) and then transcribed into cDNA through SuperScript™ III First-Strand Synthesis SuperMix (#18080400, Invitrogen, USA). The qPCR was performed using TB Green Premix Ex Taq (#RR420Q, Takara, Japan) on an ABI StepOne Plus Real-Time PCR System. The human LDHA primers used were as follow: forward 5′-TTGACCTACGTGGCTTGGAAG-3′, and reverse 5′- GGTAACGGAATCGGGCTGAAT-3′. The human GAPDH primers were as follow: forward 5′- ACAACTTTGGTATCGTGGAAGG-3′, and reverse 5′-GCCATCACGCCACAGTTTC-3′. The LDHA levels were normalized to GAPDH, and the whole experiments were repeated triplicate.

### 2.4 LDHA-related genes and enrichment analysis in EC

The LDHA-related genes were identified with *P* < 0.05 and |R| >0.3 according to the TCGA-UCEC cohort in the LinkedOmics database (https://www.linkedomics.org/login.php). The gene ontology (GO) analysis and Kyoto Encyclopedia of Genes and Genomes (KEGG) pathway analysis of LDHA-related genes were conducted by the R package “clusterProfiler” and then visualized by the “ggplot2” package.

### 2.5 Gene set enrichment analysis

The tissues from the TCGA-UCEC cohort were divided into the high LDHA group and the low LDHA group, and the two groups were analyzed through the GSEA method using the R package “clusterProfiler”. The MSigDB Collections was utilized as the reference gene set. The conditions of significance are set as adjust *P* < 0.05 & FDR (q value) < 0.25.

### 2.6 Tumor-infiltrating lymphocyteanalysis

A standardized EC dataset was downloaded from the UCSC database (https://xenabrowser.net/), and stromal, immune, and Estimation of Stromal and Immune cells in MAlignant Tumour tissues using Expression data (ESTIMATE) scores were calculated for each patient based on gene expression using the R software package “ESTIMATE”. The association between LDHA and lymphocyte infiltration levels was confirmed by the TIMER 2.0 database (http://timer.cistrome.org/). The tissue samples of TCGA-UCEC were classified into the high LDHA group and low LDHA group according to median LDHA level, and the difference in immune cells between the two groups was analyzed by the R package “stats”. The connection of LDHA mutation to TILs and the prognostic value of LDHA and TILs was explored by the TIMER 2.0 database.

### 2.7 Analysis of the relationship between LDHA and m6A modification

The correlation between LDHA and m6A-related genes was investigated in the TCGA-UCEC cohort and GSE106191 dataset by Pearson correlation analysis. The tissue samples of TCGA-UCEC were classified into the high LDHA group and low LDHA group according to median LDHA level, and the difference of m6A-related genes between the two groups was analyzed by the R package “stats”.

### 2.8 Analysis of the relationship between LDHA and ferroptosis

The correlation between LDHA and ferroptosis-related genes was investigated in the TCGA-UCEC cohort and GSE106191 dataset by Pearson correlation analysis. The tissue samples of TCGA-UCEC were classified into high LDHA group and low LDHA group according to median LDHA level, and the difference of ferroptosis-related genes between two groups was analyzed by the R package “stats”.

### 2.9 LDHA knockdown in EC cells

The shRNA targeting LDHA and the control vector were constructed by Miaoling Biology (Wuhan, China). The plasmids were transfected into EC cells using Lipofectamine 3000 Transfection Reagent (#L3000001, Invitrogen, USA) according to the manufacture. The cells were utilized for the next experiments after 48 h.

### 2.10 Western blot

The total protein was extracted from each cell by RIPA Lysis and Extraction Buffer (#89901, Thermo Scientific, USA). The protein was separated by sodium dodecyl sulfate polyacrylamide gel electrophoresis and transferred to polyvinylidene fluoride membrane. The primary antibody was Rabbit polyclonal Anti-LDHA antibody (1:1000, #PB10075, Boster, China) or β-actin mouse monoclonal antibody (1:2000, #AF2811, Beyotime, China). The second antibody was HRP Conjugated AffiniPure Donkey Anti-Rabbit IgG(H+L) (1:2000, #BA1061, Boster, China) or HRP Conjugated AffiniPure Donkey Anti-Mouse IgG(H+L) (1:2000, #BA1062, Boster, China).

### 2.11 CCK-8 assay

The cells of each group were planted in a 96-well plate with 5×10^3^ cells/well. After 24 h, the medium was replaced by 10% CCK-8 reagent (#CK04, Dojindo, Japan) at 0, 24, 48,72, and 96 h, and cultured in 37°C for 2 h. The OD450 of each well was detected by the Multiskan FC Microplate Photometer (Thermo Scientific, USA).

### 2.12 Cell clone formation assay

The cells of each group were planted in 6-well plate with 800 cells/well, and cultured in an incubator with 37°C and 5% CO_2_. Once the cell number in most clones reached 50, the cells were fixed with 4% paraformaldehyde for 30 min, and then stained with 0.1% crystal violet for 1 h. After rinsing and airing, the cell clones were photographed and counted.

### 2.13 Apoptosis detection

The cells were collected in a 1.5 ml tube and treated with an Annexin V-FITC apoptosis detection kit (#C1062S, Beyotime, China), according to the manufacturer. The cells were detected by a CytoFLEX flow cytometer (Beckman Coulter, USA).

### 2.14 Transwell migration assay

The cells were resuspended in medium without FBS, and then planted in the Transwell chamber with 1×10^4^/chamber. The chambers were placed in the 24-well plate, which contained a culture medium with 20% FBS. After 24 h, the cells on the inner membrane of the chamber were wiped gently by swabs, fixed with 4% paraformaldehyde for 30 min, and then stained with 0.1% crystal violet for 1 h. After rinsing and airing, the cells across the membrane were photographed and counted.

### 2.15 Transwell invasion assay

The Matrigel was coated on the inner membrane of the chamber and placed at 37°C until solidification. The following steps were the same as the migration assay.

### 2.16 Statistical analysis

The data *in vitro* tests were statistically analyzed using IBM SPSS Statistics v26 (Endicott, New York, USA). Measurement data were expressed as mean ± standard deviation, and the SNK-q test was used to compare the differences between the two groups. *P* < 0.05 was considered statistically significant.

## 3 Results

### 3.1 LDHA expression in patients with EC

**Figure 1:**
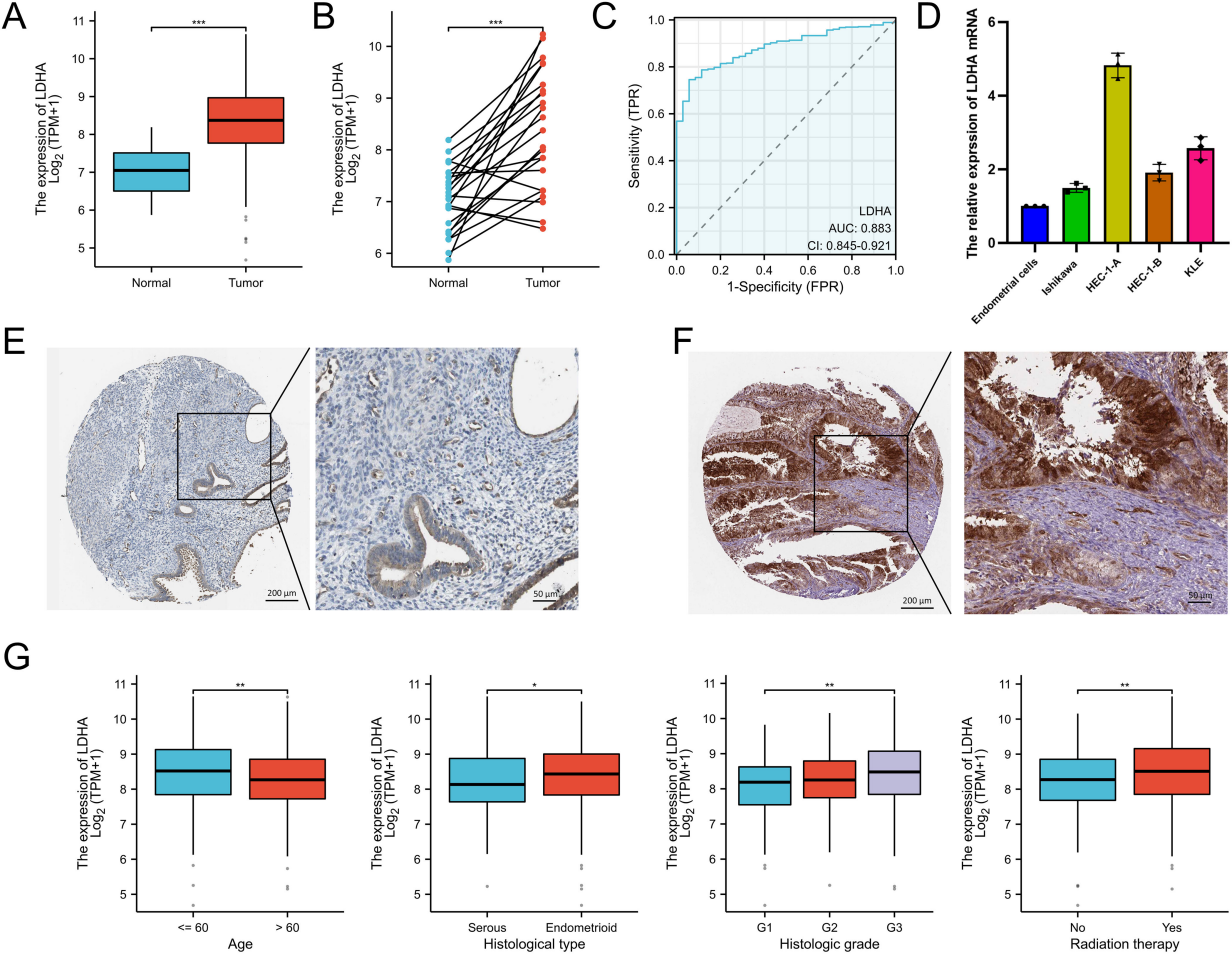
The LDHA expression in endometrial cancer. **(A)** The LDHA expression summarized in the TCGA-UCEC cohort. **(B)** LDHA expression in paired tumor/normal EC tissues based on TCGA-UCEC cohort. **(C)** ROC curve analysis of LDHA. **(D)** The LDHA mRNA in endometrial cells detected by qRT-PCR. **(E)** LDHA protein stained in normal endometrial tissues by the HPA database. **(F)** LDHA protein stained in EC tissues by the HPA database. **(G)** The relationship between LDHA and clinicopathologic features. *P<0.05, **P<0.01, *** *P*<0.001.

The TCGA data showed that the LDHA mRNA level in EC tissues was overexpressed compared to that in normal endometrial tissues (**Figures 1A, B**). Meanwhile, LDHA accurately distinguished the EC and normal tissues in the ROC curve (AUC =0.883, 95% CI: 0.845-0.921) (**Figure 1C**). The qPCR results demonstrated that LDHA mRNA expression in endometrial cells was less than in EC cells (**Figure 1D**). Furthermore, high LDHA protein levels in in EC tissues were more common than that in normal endometrial tissues, according to the data from the Human Protein Atlas database (**Figures 1E, F**). These results suggested that LDHA was upregulated in EC tissues.

To determine the clinical value of LDHA in EC patients, we analysed the relationships between LDHA and clinicopathologic features. As shown in **Figure 1G** and **Supplementary Table S1**, EC patients aged more than 60 years had lower LDHA levels than the other patients. LDHA expression in endometrioid EC patients was higher than in serous EC patients. Patients in G3 grade had the highest LDHA level, fewer in G2, and the minimum in G1. EC patients with radiation therapy had higher LDHA expression than patients without radiation therapy.

### 3.2 GO and KEGG enrichment pathways of LDHA-related genes

**Figure 2:**
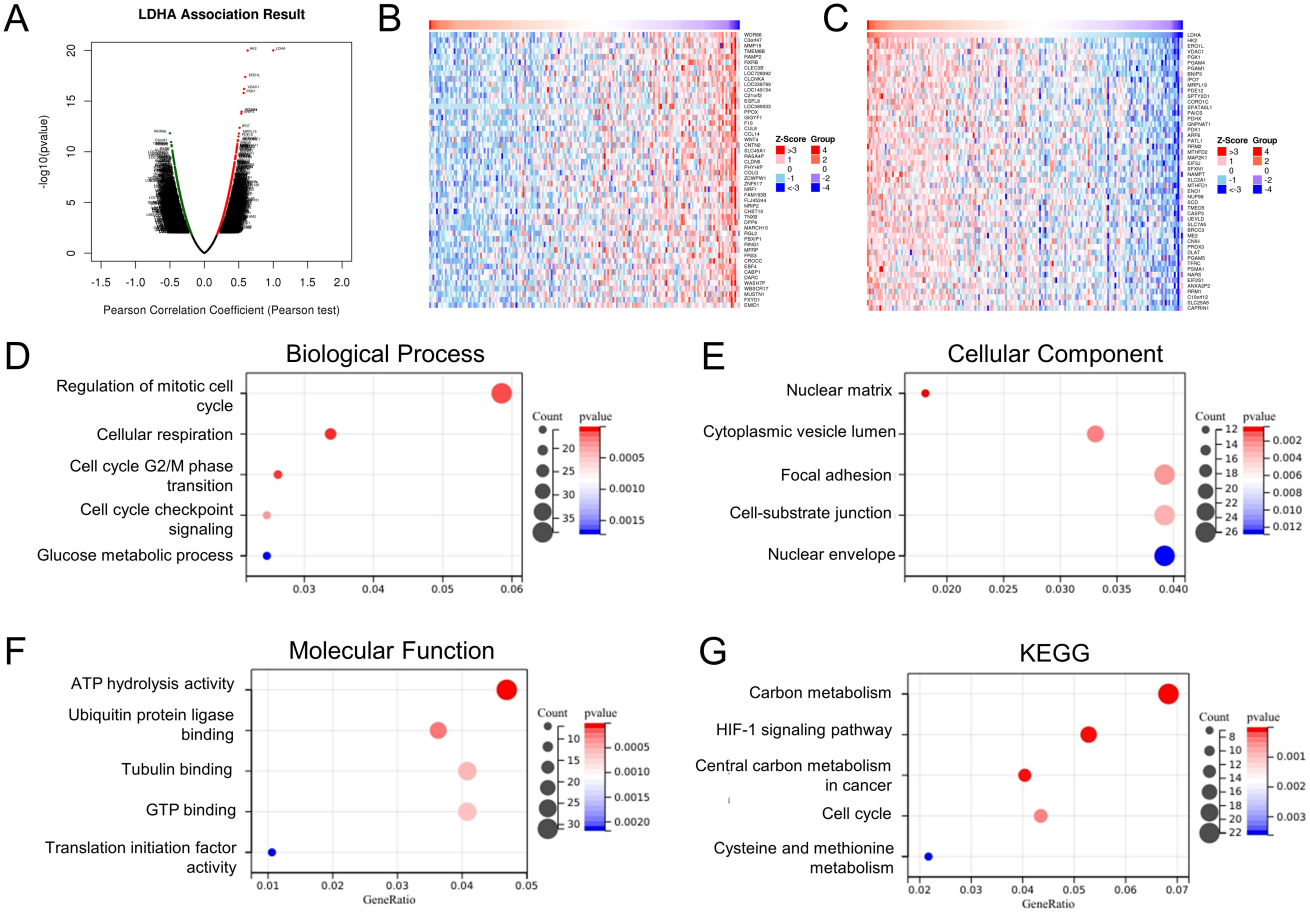
Enrichment analysis of LDHA-related genes in EC. **(A)** LDHA-related genes in TCGA-UCEC cohort detected by the LinkedOmics database. The top 50 co-expression genes positively **(B)** and negatively **(C)** associated with LDHA in the TCGA-UCEC cohort. **(D–F)** Enrichment analysis of (GO) terms for LDHA-related genes. **(G)** Enrichment analysis of KEGG terms for LDHA-related genes.

To further explore the function and mechanism of LDHA in EC tissues, we determined the co-expressed genes associated with LDHA expression in the TCGA-UCEC dataset via the LinkedOmics database (**Figures 2A–C**). The GO analysis showed that, in the biological process group, LDHA-related genes mainly enriched in the regulation of the mitotic cell cycle, cellular respiration, cell cycle G2/M phase transition, cell cycle checkpoint signaling, and glucose metabolic process (**Figure 2D**). In the cellular component group, LDHA-related genes are mainly enriched in the nuclear matrix, cytoplasmic vesicle lumen, focal adhesion, cell-substrate junction, and nuclear envelope (**Figure 2E**). In the molecular function group, LDHA-related genes are mainly enriched in ATP hydrolysis activity, ubiquitin protein ligase binding, tubulin binding, GTP binding, and translation initiation factor activity (**Figure 2F**). The KEGG pathway analysis confirmed that these genes involved carbon metabolism, HIF-1 signaling pathway, central carbon metabolism in cancer, cell cycle, cysteine, and methionine metabolism (**Figure 2G**). These results suggested that LDHA might regulate cell cycle and metastasis in EC progression.

### 3.3 Gene set enrichment analysis of LDHA-related genes

**Figure 3:**
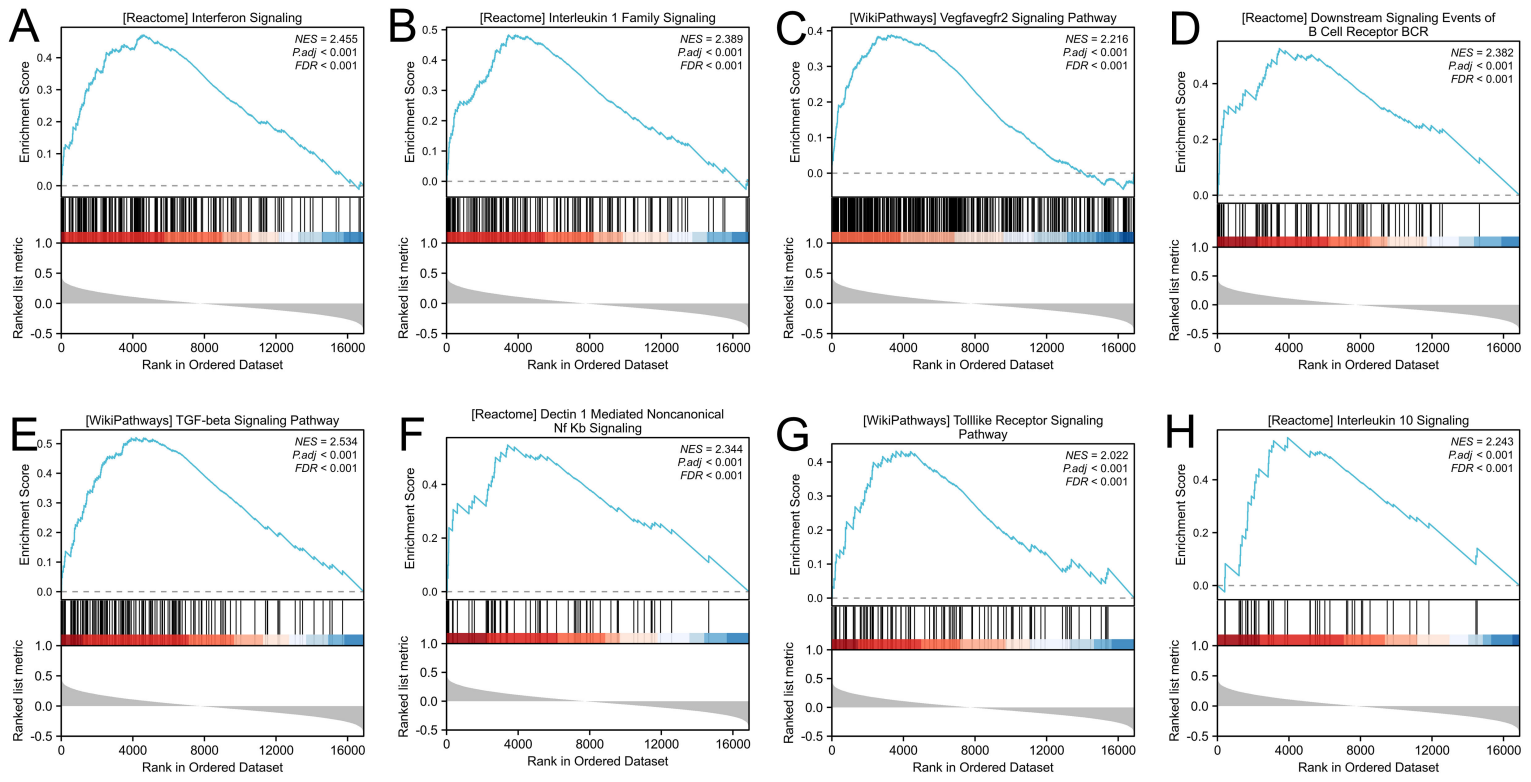
Gene set enrichment analysis. **(A)** Interferon signaling. **(B)** Interleukin 1 family signaling. **(C)** VEGFA-VEGFR2 signaling. **(D)** Downstream signaling events of B cell receptor. **(E)** TGF-beta signaling pathway. **(F)** Dectin1 mediated noncanonical NF-κB signaling. **(G)** Toll-like receptor signaling pathway. **(H)** Interleukin 10 signaling.

We performed GSEA on LDHA-related genes to investigate the mechanism of LDHA in EC progression. The results demonstrated that enrichment pathways included interferon signaling (FDR<0.001, *P*<0.001), interleukin 1 family signaling (FDR<0.001, *P*<0.001), VEGFA-VEGFR2 signaling (FDR<0.001, *P*<0.001), downstream signaling events of B cell receptor (FDR<0.001, *P*<0.001), TGF-beta signaling pathway (FDR<0.001, *P*<0.001), Dectin1 mediated noncanonical NF-κB signaling (FDR<0.001, *P*<0.001), Toll-like receptor signaling pathway (FDR<0.001, *P*<0.001), and interleukin 10 signaling (FDR<0.001, *P*<0.001) (**Figure 3**). These results show that LDHA in EC tissues were likely to regulate the immune cells and immune microenvironment.

### 3.4 Influence of LDHA interference on the malignant biological behavior of EC cells

**Figure 4:**
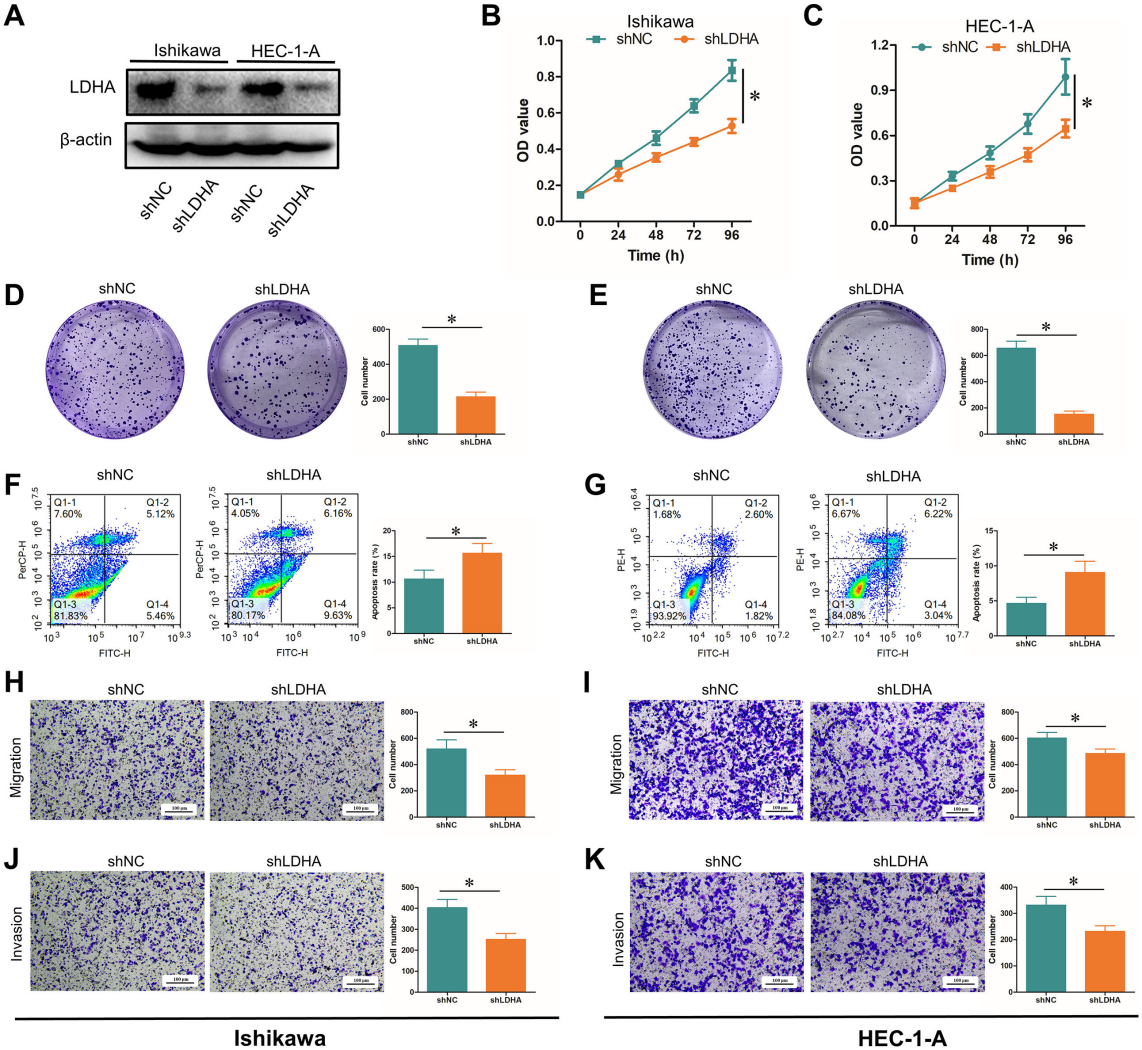
Influence of LDHA knockdown on EC functions. **(A)** LDHA protein level after LDHA knockdown detected by Western Blot. **(B, C)** Cell proliferation after LDHA knockdown detected by CCK-8 assay. **(D, E)** Cell proliferation after LDHA knockdown detected by cell clone formation assay. **(F, G)**. Cell apoptosis after LDHA knockdown detected by flow cytometry. **(H, I)** Cell migration after LDHA knockdown was detected by the Transwell assay. **(J, K)** Cell invasion after LDHA knockdown was detected by the Transwell assay. *P<0.05.

To further identify the role of LDHA in EC progression, we downregulated the LDHA expression in EC cell lines Ishikawa and HEC-1-A (**Figure 4A**). CCK-8 assay and cell clone formation assay confirmed that LDHA knockdown inhibited the proliferation of EC cells (**Figures 4B–E**). Flow cytometry demonstrated that that LDHA downregulation promoted EC cell apoptosis (**Figures 4F, G**). Transwell assay verified that LDHA interference decreased the migration and invasion of EC cells (**Figures 4H–K**). These results suggested that the downregulation of LDHA could restrain the malignant biological behavior of EC cells.

### 3.5 Connection of LDHA expression to TILs

**Figure 5:**
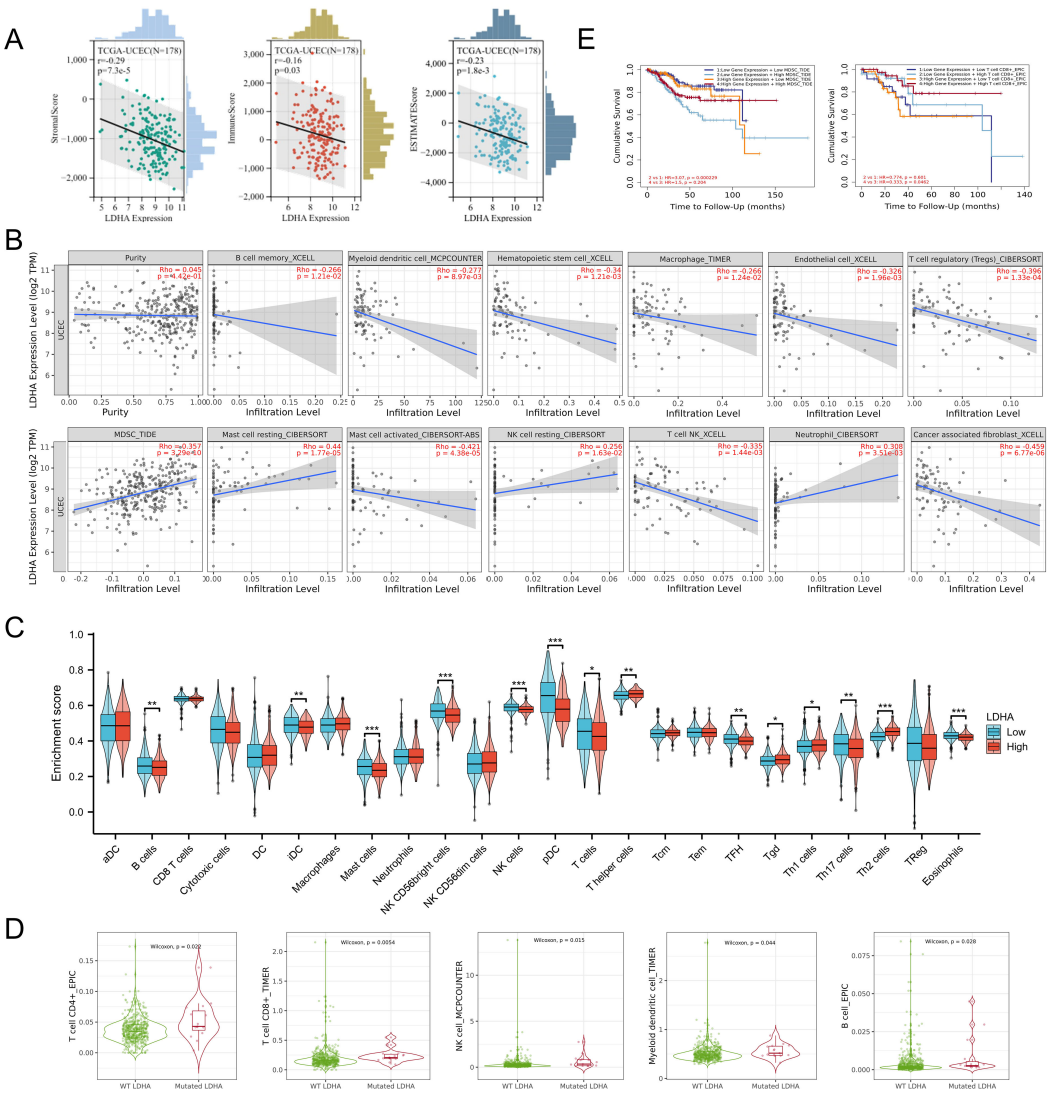
Associations between LDHA and tumor immune infiltrating cells. **(A)** Correlation of LDHA to stromal cells and immune cells calculated by the ESTIMATE method. **(B)** Relationship between LDHA expression and infiltration levels of immune cells. **(C)** Enrichment scores of immune cells in the high LDHA group and low LDHA group. **(D)** Infiltration levels of immune cells in WT LDHA group and mutated LDHA group. **(E)** The survival curves of patients with different combinations of LDHA and immune cells. WT, wild type. *P<0.05, **P<0.01, *** *P*<0.001.

The association between LDHA expression and cancer microenvironment was first evaluated by the ESTIMATE method. As shown in **Figure 5A**, LDHA level was negatively connected to StromalScore, ImmuneScore, and ESTIMATEScore. The relationship between LDHA expression and immune cells was analyzed through the TIMER 2.0 database. The results confirmed that LDHA expression level was negatively related to memory B cell, myeloid dendritic cell (DC), hematopoietic stem cell, macrophage, endothelial cell, Tregs, activated mast cell, NK T cell, and cancer-associated fibroblast, and positively associated with myeloid-derived suppressor cells (MDSC), resting mast cell, resting NK cell, and neutrophil (**Figure 5B**).

Subsequently, the patients from the TCGA-UCEC cohort were classified into the high LDHA group and the low LDHA group, and the difference of immune cell enrichment was investigated between the two groups. The result confirmed that the high LDHA group had fewer B cells, immature DC (iDC), mast cells, NK CD56 bright cells, NK cells, plasmacytoid DC (pDC), T cells, T follicular helper (Tfh) cells, Th17 cells, and eosinophils, and more T helper cells, Tgd, and Th2 cells (**Figure 5C**). We further identified the connection of LDHA expression to TILs by analyzing the relationship between immune cell markers and LDHA level. All data from the TIMER 2.0, GEPIA, and TCGA database demonstrated that LDHA level was associated with gene markers of M1 macrophage (PTGS2), DC (NRP1), Th1 (STAT1), Tfh (IL21), Th17 (STAT3), and Treg (CCR8 and STAT5B) (**Table 1**).

**Table 1:** Associations between LDHA and gene markers of immune cells in TIMER, GEPIA, and TCGA database.

Description	Marker	TIMER		GEPIA		TCGA	
		Purity adjustment		Tumor		Tumor	
		rho	*P*	rho	*P*	rho	*P*
CD8+ T Cell	CD8A	-0.175	3.22E-01	0.076	3.20E-01	0.086	**4.40E-02**
	CD8B	-0.077	1.90E-01	-0.093	2.20E-01	-0.016	7.13E-01
T Cell (general)	CD3D	-0.063	2.79E-01	-0.120	1.10E-01	-0.044	2.97E-01
	CD3E	-0.054	3.61E-01	-0.015	8.40E-01	-0.036	4.01E-01
	CD2	-0.012	8.37E-01	-0.018	8.10E-01	-0.004	9.30E-01
B Cell	CD19	-0.175	**2.58E-03**	-0.140	6.60E-02	-0.139	**1.00E-03**
	CD79A	-0.024	6.87E-01	-0.064	4.00E-01	-0.044	3.03E-01
Monocyte	CD86	0.055	3.51E-01	0.033	6.70E-01	0.106	**1.20E-02**
	CSF1R	-0.137	**1.91E-02**	-0.047	5.40E-01	-0.053	2.09E-01
TAM	CCL2	0.059	3.12E-01	0.050	5.10E-01	0.108	1.10E-02
	CD68	0.137	**1.89E-02**	0.210	**5.80E-03**	0.064	1.32E-01
	IL10	0.063	2.84E-01	-0.006	9.40E-01	0.085	**4.4E-02**
M1	NOS2	0.062	2.88E-01	0.230	**1.80E-03**	0.068	1.1E-01
	IRF5	-0.061	3.01E-01	-0.053	4.90E-01	-0.010	8.17E-01
	PTGS2	0.211	**2.48E-04**	0.260	**5.20E-04**	0.262	**1.23E-10**
M2	CD163	0.114	5.18E-02	-0.080	2.90E-01	0.163	**2.00E-04**
	MRC1	0.154	**8.39E-03**	0.150	5.20E-02	0.145	**6.00E-04**
	MS4A4A	0.042	4.77E-01	-0.019	8.00E-01	0.077	7.10E-02
Neutrophils	CEACAM8	0.063	2.80E-01	-0.036	6.40E-01	-0.051	2.27E-01
	ITGAM	-0.068	2.44E-01	0.087	2.50E-01	-0.011	8.03E-01
	CCR7	-0.087	1.38E-01	0.030	6.9E-01	-0.086	**4.20E-02**
NK Cell	KIR2DL1	0.039	5.05E-01	-0.019	8.00E-01	0.078	6.80E-02
	KIR2DL3	0.061	2.95E-01	-0.019	8.10E-01	0.052	2.21E-01
	KIR2DL4	0.112	5.58E-02	0.083	2.80E-01	0.185	**1.13E-05**
	KIR3DL1	0.054	3.53E-01	0.043	5.80E-01	0.126	**3.00E-03**
	KIR3DL2	0.028	6.34E-01	0.120	1.30E-01	0.047	2.71E-01
	KIR2DL3	0.061	2.95E-01	-0.019	8.10E-01	0.052	2.21E-01
	KIR2DS4	0.007	9.06E-01	-0.034	6.60E-01	0.036	3.92E-01
Dendritic Cell	HLA-DQB1	-0.053	3.69E-01	-0.014	8.50E-01	-0.027	5.26E-01
	NRP1	0.170	**3.44E-03**	0.180	**1.60E-02**	0.171	**5.41E-05**
	ITGAX	-0.083	1.56E-01	0.096	2.10E-01	-0.054	2.03E-01
Th1	TBX21	-0.005	9.38E-01	0.032	6.70E-01	0.006	8.90E-01
	STAT4	-0.050	3.97E-01	0.010	8.90E-01	-0.029	5.03E-01
	STAT1	0.301	**1.47E-07**	0.370	**3.50E-07**	0.313	**6.77E-14**
	IFNG	0.166	**4.31E-03**	0.079	3.00E-01	0.185	**1.21E-05**
	TNF	0.144	**1.39E-03**	0.041	5.90E-01	0.199	**2.47E-06**
	IL12A	-0.129	**2.71E-02**	-0.076	3.20E-01	-0.037	3.80E-01
	IL12B	0.010	8.66E-01	0.031	6.80E-01	-0.010	8.08E-01
Th2	GATA3	-0.051	3.81E-01	-0.076	3.20E-01	-0.018	6.80E-01
	STAT6	0.019	7.48E-01	0.018	8.10E-01	0.144	**7.00E-04**
	STAT5A	0.006	9.22E-01	-0.004	9.60E-01	0.050	2.42E-01
	IL13	-0.060	3.03E-01	0.093	2.20E-01	-0.048	2.61E-01
Tfh	BCL6	-0.020	7.32E-01	0.2	**7.60E-03**	0.049	2.48E-01
	IL21	0.179	**2.13E-03**	0.240	**1.80E-03**	0.190	**6.35E-06**
Th17	STAT3	0.315	**3.42E-08**	0.510	**7.9E-13**	0.376	**2.30E-27**
	IL17A	0.109	6.23E-02	-0.009	9.00E-01	0.084	4.90E-02
Treg	FOXP3	0.033	5.73E-01	0.022	7.70E-01	0.001	9.99E-01
	CCR8	0.122	**3.61E-02**	0.170	**2.90E-02**	0.138	**1.00E-03**
	STAT5B	0.173	**2.98E-03**	0.270	**2.50E-04**	0.204	**1.34E-06**
	TGFB1	0.029	6.23E-01	0.080	3.00E-01	0.111	**9.20E-03**
T cell exhaustion	PDCD1	-0.026	6.59E-01	-0.018	8.10E-01	0.002	9.64E-01
	CTLA4	0.023	6.96E-01	-0.005	9.50E-01	0.038	3.71E-01
	LAG3	0.087	1.36E-01	-0.082	2.80E-01	0.117	**5.80E-03**
	HAVCR2	0.064	2.72E-01	0.054	4.80E-01	0.121	**4.40E-03**
	GZMB	0.133	**2.32E-02**	-0.028	7.10E-01	0.154	**3.00E-04**

Bold values for P < 0.05.

LDHA mutation was also confirmed to relate to immune cells via TIMER 2.0. The mutated LDHA group had more CD4^+^ T cells, CD8^+^ T cells, NK cells and myeloid DCs (**Figure 5D**). In addition, LDHA combined with immune cells could affect the survival of EC patients. In patients with low LDHA expression, high MDSC levels tended to forebode a poor prognosis, but this phenomenon disappeared in patients with high LDHA expression (**Figure 5E**). The level of CD8^+^ T cells only affected the survival of EC patients with high LDHA expression (**Figure 5E**). The results above suggested that LDHA was significantly associated with infiltrating lymphocytes in the tumor microenvironment.

### 3.6 Connection of LDHA expression to ferroptosis in EC

**Figure 6:**
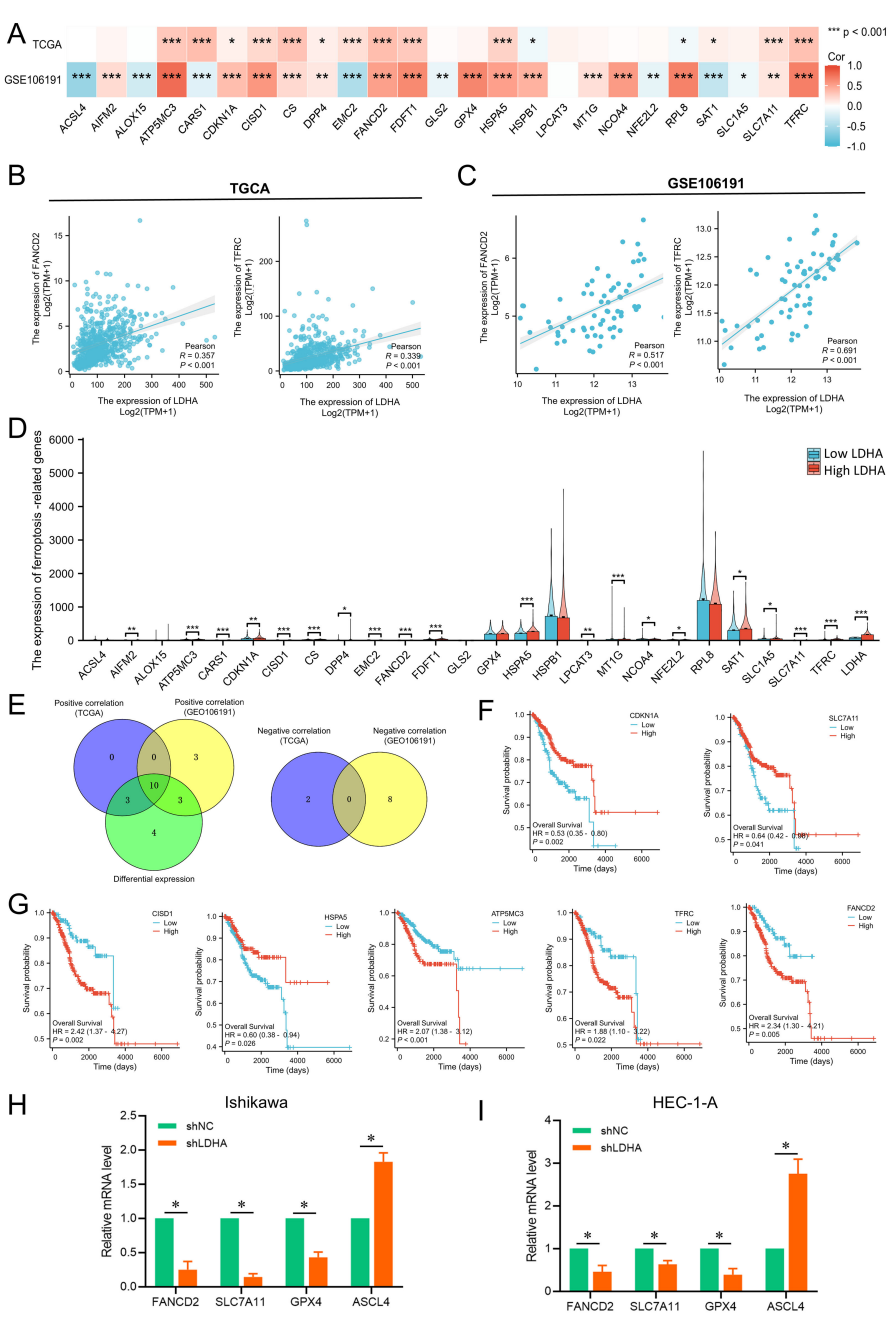
Associations between LDHA expression and ferroptosis-related genes in EC. **(A)** Connection of LDHA to ferroptosis-related genes in GSE106191 and TCGA-UCEC cohort. **(B)** Connection of LDHA to FANCD2 and TFRC in TCGA-UCEC cohort. **(C)** Connection of LDHA to FANCD2 and TFRC in GSE106191. **(D)** The differential expression of ferroptosis-related genes between high and low LDHA groups in the TCGA-UCEC cohort. **(E)** Hub genes of expression association and differential expression. **(F, G)** The Kaplan–Meier curve of hub genes. **(H, I)** The changes of ferroptosis-related genes after LDHA knockdown in EC cells. *P<0.05, **P<0.01, *** *P*<0.001.

Ferroptosis has been confirmed to regulate EC progression. This study explored the relationship between LDHA and ferroptosis through TCGA and GSE106191. In TCGA-UCEC cohort, LDHA expression was positively connected to ferroptosis related genes like ATP5MC3, CARS1, CDKN1A, CISD1, CS, DPP4, EMC2, FANCD2, FDFT1, HSPA5, SAT1, SLC7A11, and TFRC, and negatively related to HSPB1 and RPL8 (**Figure 6A**). In GSE106191, LDHA expression was positively associated with AIFM2, ATP5MC3, CDKN1A, CISD1, CS, DPP4, FANCD2, FDFT1, GPX4, HSPA5, HSPB1, MT1G, NCOA4, RPL8, SLC7A11, and TFRC, and negatively related to ACSL4, ALOX15, CARS1, EMC2, GLS2, NFE2L2, SAT1, and SLC1A5 (**Figure 6A**). Among these genes, FANCD2 and TFRC were the top relative genes with weak correlation (R=0.2-0.4) in the TCGA cohort, but with a moderate correlation (R=0.4-0.6) and a high correlation (R=0.6-0.8) respectively in the GSE106191 (**Figures 6B, C**).

Then, the samples of TCGA-UCEC were classified into the high LDHA group and low LDHA group, and the difference of 25 ferroptosis-related genes in the two groups was analyzed. As shown in **Figure 6D**, high LDHA group had higher levels of AIFM2, ATP5MC3, CARS1, CDKN1A, CISD1, CS, PDD4, EMC2, FANCD2, FDFT1, HSPA5, LPCAT3, MT1G, NCOA4, NFE2L2, SAT1, SLC1A5, SLC7A11, and TFRC. A total of 10 genes were identified as key genes for LDHA expression correlation and differential expression relationship, including CDKN1A, DPP4, SLC7A11, CISD1, HSPA5, FDFT1, ATP5MC3, CS, TFRC and FANCD2 (**Figure 6E**). Furthermore, the expressions of CDKN1A, SLC7A11, CISD1, HSPA5, ATP5MC3, TFRC, and FANCD2 significantly affect the overall survival of EC patients (**Figures 6F, G**). Finally, LDHA knockdown in EC cells could significantly decrease the mRNA levels of FANCD2, SLC7A11 and GPX4, and increase the ASCL4 expression (**Figures 6H, I**). These results indicated that LDHA might regulate the survival and progression of EC by impacting the ferroptosis.

### 3.7 Connection of LDHA expression to m6A modification in EC

**Figure 7:**
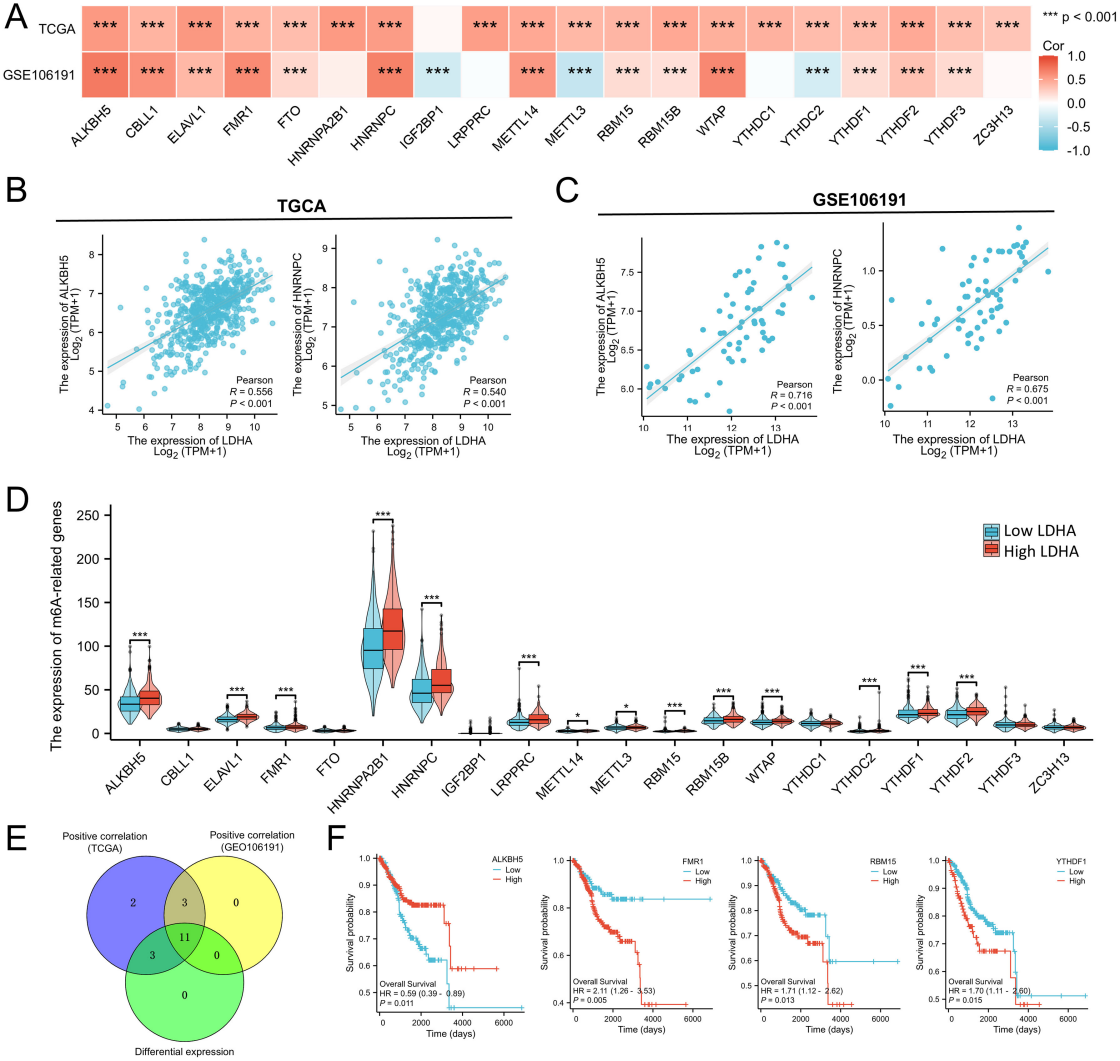
Associations between LDHA expression and m6A-related genes in EC. **(A)** Connection of LDHA to m6A-related genes in GSE106191 and TCGA-UCEC cohort. **(B)** Connection of LDHA to ALKBH5 and HNRNPC in TCGA-UCEC cohort. **(C)** Connection of LDHA to ALKBH5 and HNRNPC in GSE106191. **(D)** The differential expression of m6A-related genes between high and low LDHA groups in the TCGA-UCEC cohort. **(E)** Hub genes of expression association and differential expression. **(F)** The Kaplan–Meier curve of hub genes. *P<0.05, *** *P*<0.001.

The m6A modification also plays a vital role in EC progression. We analyzed the relationship between LDHA level and 20 key genes of m6A modification based on the TCGA-UCEC and GSE106191 dataset. As shown in **Figure 7A**, LDHA expression was positively associated with almost all m6A-related genes except IGF2BP1 in the TCGA cohort. In GSE106191 dataset, LDHA level was positively connected to ALKBH5, CBLL1, ELAVL1, FMR1, FTO, HNRNPC, METTL14, RBM15, RBM15B, WTAP, YTHDF1, YTHDF2, and YTHDF3, and negatively related to IGF2BP1, METTL3, and YTHDC2 (**Figure 7A**). Among these genes, ALKBH5 and HNRNPC were the top relative genes with moderate correlations in both the TCGA cohort and with high correlations in the GSE106191 (**Figures 7B, C**).

Then, the samples of TCGA-UCEC were classified into high LDHA group and low LDHA group, and the difference of 20 m6A-related genes in two groups was analyzed. As shown in **Figure 7D**, high LDHA group had higher levels of ALKBH5, ELAVL1, FMR1, HNRNPA2B1, HNRNPC, LRPPRC, METTL14, METTL3, RBM15, RBM15B, WTAP, YTHDC2, YTHDF1, and YTHDF2. A total of 11 genes were identified as key genes for LDHA expression correlation and differential expression relationship, including ALKBH5, ELAVL1, FMR1, HNRNPC, METTL14, RBM15, RBM15B, WTAP, YTHDC2, YTHDF1, YTHDF2 (**Figure 7E**). Furthermore, the expressions of ALKBH5, FMR1, RBM15, and YTHDF1 significantly affect the overall survival of EC patients (**Figure 7F**). These results indicated that LDHA might affect the prognosis and progression of EC by regulating the m6A modification.

## 4 Discussion

The enhancement of aerobic glycolysis is a significant feature of the cancer metabolic process (9). The process of glycolysis is the conversion of glucose to pyruvate, which eventually produces lactic acid (10). In this process, LDHA is a crucial player in ATP production and regeneration of oxidized NAD necessary for proliferation and invasion of tumor cells (11). Studies have confirmed the overexpression of LDHA in renal cell carcinoma (12), oral squamous cell carcinoma (13), cervical cancer (14), lung adenocarcinoma (15, 16), and cholangiocarcinoma (17). LDHA seems to be a molecular target in various cancer therapies, but little is known about the function of LDHA in EC progression.

In this study, we explored the LDHA expression in EC tissues through the TCGA database and HPA database and found that both mRNA and protein levels of LDHA were upregulated in EC tissues compared to normal endometrial tissues, similar to the findings in other cancers (12–17). In addition, the LDHA mRNA in human EC cell lines was overexpressed than in endometrial cells. The ROC curve was constructed to calculate the diagnosis value of LDHA in EC, and we found that LDHA had a high accuracy in distinguishing the normal and EC tissues. These results suggested that high LDHA level in endometrial tissues might be a biomarker for EC diagnosis. The relationships between LDHA level and clinicopathologic features have been found in different cancers (18, 19). We also found that LDHA expression was associated with age, histological type, histologic grade, and radiation therapy. Especially, EC patients with higher histologic grade had a higher level of LDHA expression, which meant that LDHA level may relate to the histologic grade and then affect the EC prognosis. These results suggested that LDHA might be a diagnostic and prognostic biomarker for EC patients.

Most previous research about LDHA focuses on energy metabolism and glycolysis, and other biological functions and signaling pathways may be ignored. We determined the LDHA co-expression genes and explored the potential mechanism of LDHA in EC. The GO analysis showed that except for energy and glucose metabolism, LDHA-related genes mainly enriched in the cell cycle, nuclear matrix, focal adhesion, cell-substrate junction, and tubulin binding. KEGG analysis demonstrated that LDHA was connected to the HIF-1 signaling pathway and cell cycle. These results indicated LDHA might be involved in EC cell proliferation, migration and invasion. Hou et al. (20) found that LDHA promoted the proliferation, migration and invasion of thyroid cancer cells. Forkasiewicz et al. (21) demonstrated that LDHA can regulate TNF-α-induced esophageal cancer cell migration through the ERK1/2 signaling pathway. In a breast cancer model, LDHA knockdown can weaken glycolysis and significantly reduce tumor growth by affecting mitochondrial physiology (22). In glioblastoma, silencing LDHA expression leads to reduced glycolysis, slower cell growth, increased apoptosis, and reduced invasion (23). To verify our hypothesis, we downregulated the LDHA expression in EC cell lines. We found that LDHA inference inhibited the proliferation, migration, and invasion of EC cells and promoted cell apoptosis. Our results are similar to those reported in other cancers, and suggested that LDHA could promote malignant biological behavior of EC cells. Therefore, targeting LDHA in EC cells had the potential to become a novel therapeutic strategy for EC patients.

The GSEA demonstrated that LDHA co-expression genes were enriched in interferon signaling, interleukin 1 family signaling, downstream signaling events of B cell receptor, Toll-like receptor signaling pathway, and interleukin 10 signaling, which meant LDHA might participate in regulating the immune microenvironment. Recent studies have shown that immunotherapy achieved satisfactory therapeutic effects in patients with advanced, recurrent, and metastatic EC, and there are more potential tumor therapeutic targets to be explored (24, 25). Therefore, it’s helpful to investigate the immune regulation mechanism in the immune microenvironment the to diagnose and treat EC patients. Tumor cells could change the intracellular metabolic environment and regulate the function of immune cells by enhancing glycolytic metabolism. As a vital regulator of glycolysis, LDHA has the potential to affect the immune microenvironment. The ESTIMATE is a method to infer the contents of tumor cells and the different infiltrating normal cells by utilizing the unique nature of the transcription profile of cancer samples (26). We conducted the ESTIMATE method and found that LDHA was negatively connected to stromal cells and immune cells in the tumor microenvironment. In the TIMER2.0 database, LDHA expression level was negatively related to the infiltration levels of memory B cell, myeloid DC, activated mast cell, and NK T cell, which inhibit the tumor progression and benefit patients’ prognosis (27–31). Simultaneously, LDHA was positively associated with the infiltration levels of MDSC, resting mast cell, and resting NK cell that induces immunosuppressive tumor microenvironment and tumor immune escape (32, 33). The analysis of TCGA-cohort also acquired similar results, and LDHA expression showed positive connection to B cells, immature iDC, mast cells, NK cells, pDC, T cells, Tfh cells, Th17 cells, and eosinophils, and negative relation to T helper cells, Tgd, and Th2 cells. LDHA mutation was related to more CD4^+^ T cells, CD8^+^ T cells, NK cells, and myeloid DCs compared to wild type, which meant LDHA might decrease infiltrations of these immune cells. In addition, LDHA level also influences the prognostic value of MDSC and CD8^+^ T cell. In patients with low LDHA expression, high MDSC level tended to predict poor prognosis, while MDSC level had no significant effect on survival in patients with high LDHA expression. In patients with high LDHA expression, low CD8^+^ T cell was connected to poor prognosis, but the prognostic role of CD8^+^ T cell disappeared in patients with low LDHA expression. These results demonstrated that LDHA level was associated with infiltration levels of some immune cells, especially B cells, DC, and MDSC. Of course, the close relationship between LDHA and immune cells empowered LDHA to become a new candidate in EC immunotherapy. Nevertheless, more research is encouraged to verify our findings more accurately.

Cancer cells need more iron than normal cells as they grow, making them more sensitive to iron-mediated necrosis (34). Numerous studies about ferroptosis provide new insights into the occurrence, metastasis, recurrence, treatment, and prognosis evaluation of EC (35–37). At the same time, glycolysis shows great influence on ferroptosis regulation. LDHA might inhibit the ferroptosis through regulating glycolysis and energy metabolism, then promoting the EC progression. We explored the correlation of LDHA to ferroptosis-related genes in the TCGA-UCEC cohort and GSE106191 and confirmed that LDHA showed a significant correlation to most ferroptosis-related genes, among which FANCD2 and TFRC were top genes in both datasets. FANCD2 was confirmed to be upregulated in EC tissues, and the knockdown of FANCD2 could inhibit the proliferation and migration of EC cells (38). TFRC was identified as a prognostic biomarker and could promote tumor progression in multiple cancers (39–41). Subsequently, we found 19 of 25 ferroptosis-related genes in the high LDHA group upregulated compared to the low LDHA group. The hub genes of the above three analyses included 10 members, and 7 genes were associated with overall survival according to the Kaplan–Meier survival analysis. Of course, FANCD2 and TFRC were embraced. Finally, we found that LDHA knockdown in EC cells could change the mRNA expressions of FANCD2, SLC7A11, GPX4 and ASCL4, just similar to findings above. These results suggested that LDHA might regulate EC progression through affecting these ferroptosis-related genes.

N6-methyladenosine (m6A) modification is the most abundant epigenetic modification in RNA and participates in EC pathogenesis and progression (42). IGF2BP1, HNRNPA2B1, FTO, WTAP promote the EC progression (43–46), but METTL3, METTL14, YTHDF2 work as tumor suppressors in EC (47–49). These m6A enzymes could regulate the glycolysis through multiple pathways, and we wondered if the LDHA level in EC could be regulated by the m6A modification. We explored the correlation of LDHA to m6A-related genes in the TCGA-UCEC cohort and GSE106191 and confirmed that LDHA showed a significant correlation to most m6A-related genes. In the high LDHA group, we found 14 of 20 m6A-related genes were upregulated compared to the low LDHA group. The hub genes of the above three analyses included 11 members, and 4 genes were associated with overall survival according to the Kaplan–Meier survival analysis, including ALKBH5, FMR1, RBM15, and YTHDF1. ALKBH5 has been demonstrated to promote the proliferation and invasion of EC cells (50). FMR1 overexpressed in colorectal cancer tissues and enhanced the proliferation and metastasis of tumors (51). RBM15 interference in cervical cancer cells could suppress the proliferation, invasion, and migration *in vitro* and *in vivo* (52). YTHDF1 facilitated cell proliferation in colorectal cancer cell lines and primary organoids, as well as lung and liver metastasis *in vivo* (53). The results above suggested that LDHA might regulate EC progression and prognosis by interacting with key m6A enzymes.

In conclusion, LDHA was upregulated in EC and connected to clinicopathologic features. Knockdown of LDHA could suppress the malignant biological behavior of EC cells, and LDHA expression related to the infiltration level of immune cells. LDHA also affected the m6A modification and ferroptosis which regulated EC progression. LDHA has the potential to become a biomarker for the diagnosis and treatment of EC patients. However, the relationship between LDHA and immune cells, m6A modification, and ferroptosis needs further validation by more experiments.

## Data Availability

The original contributions presented in the study are included in the article/**Supplementary Material**. Further inquiries can be directed to the corresponding author/s.
