# Supplementary material for: Lactate dehydrogenase A is a diagnostic biomarker associated with immune infiltration, m6A modification and ferroptosis in endometrial cancer
Source: Front Oncol. 2024 Nov 8;14:1458344. doi: 10.3389/fonc.2024.1458344 (PMC11581964; doi:10.3389/fonc.2024.1458344)
Supplement: Supplementary file 1 [file Table1.docx]

Supplementary Material

Supplementary Table 1 Associations between LDHA expression and clinicopathological characteristics in EC patients

| Characteristics | LDHA expression, n (%) | | Pearson χ2 | P |
| --- | --- | --- | --- | --- |
|  | Low | High |  |  |
| Age |  |  | 6.677 | 0.010* |
| <= 60 | 89 (43.00) | 118 (57.00) |  |  |
| > 60 | 187 (54.36) | 157 (45.64) |  |  |
| Race |  |  | 0.107 | 0.744 |
| Black or African American | 54 (49.54) | 55 (50.46) |  |  |
| White | 195 (51.32) | 185 (48.68) |  |  |
| BMI |  |  | 0.861 | 0.353 |
| <= 30 | 101 (47.64) | 111 (52.36) |  |  |
| > 30 | 160 (51.78) | 149 (48.22) |  |  |
| Menopause status |  |  | 0.269 | 0.874 |
| Pre | 16 (45.71) | 19 (54.29) |  |  |
| Peri | 8 (47.06) | 9 (52.94) |  |  |
| Post | 227 (49.89) | 228 (50.11) |  |  |
| Histological type |  |  | 7.504 | 0.023* |
| Endometrioid | 192 (46.60) | 220 (53.40) |  |  |
| Serous | 70 (59.32) | 48 (40.68) |  |  |
| Mixed | 15 (62.50) | 9 (37.50) |  |  |
| Histologic grade |  |  | 5.482 | 0.019* |
| G1& G2 | 124 (56.36) | 96 (43.64) |  |  |
| G3 | 149 (46.13) | 174 (53.87) |  |  |
| Tumor invasion (%) |  |  | 1.712 | 0.190 |
| < 50 | 142 (54.41) | 119 (45.59) |  |  |
| >= 50 | 104 (48.37) | 111 (51.63) |  |  |
| Clinical stage |  |  | 0.432 | 0.511 |
| I & II | 201 (50.89) | 194 (49.11) |  |  |
| III & IV | 76 (47.80) | 83 (52.20) |  |  |
| Residual tumor |  |  | 0.029 | 0.985 |
| R0 | 194 (51.46) | 183 (48.54) |  |  |
| R1 | 11 (50.00) | 11 (50.00) |  |  |
| R2 | 8 (50.00) | 8 (50.00) |  |  |
| Hormones therapy |  |  | 0.298 | 1.083 |
| No | 141 (47.16) | 158 (52.84) |  |  |
| Yes | 26 (55.32) | 21 (44.68) |  |  |
| Radiation therapy, |  |  | 5.754 | 0.016* |
| No | 154 (54.80) | 127 (45.20) |  |  |
| Yes | 110 (44.35) | 138 (55.65) |  |  |

**P*＜0.05.
